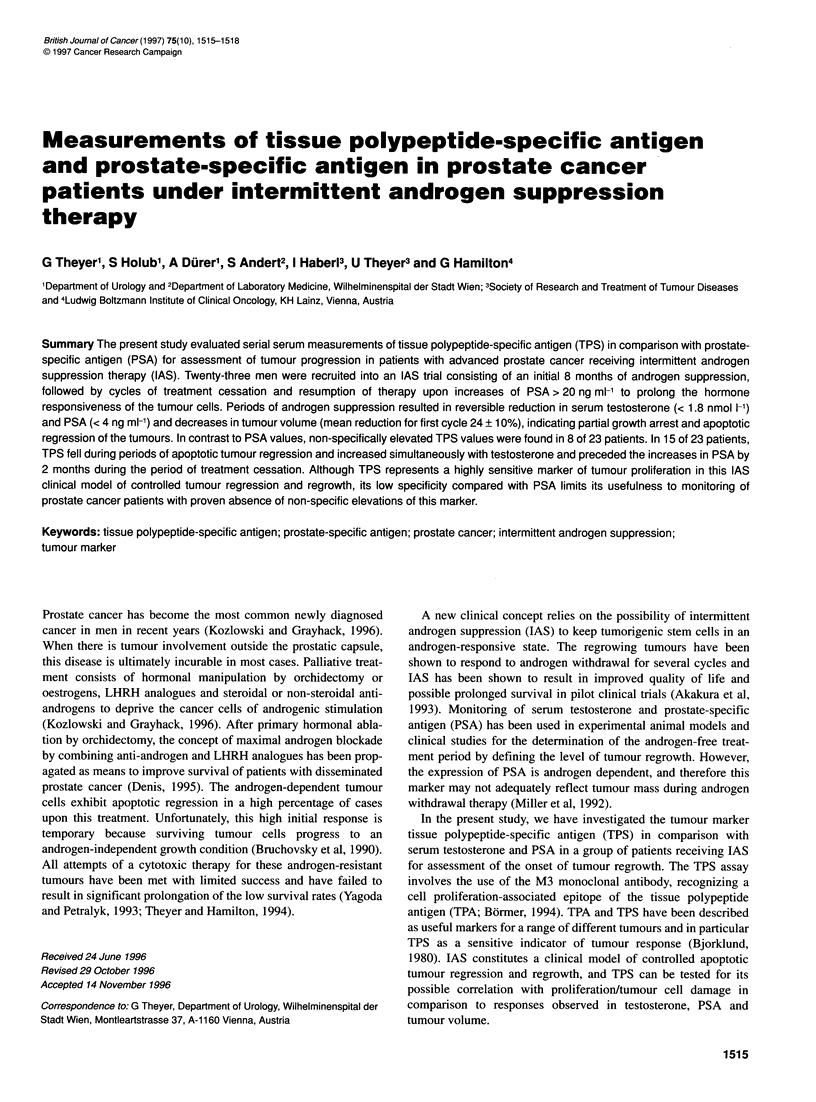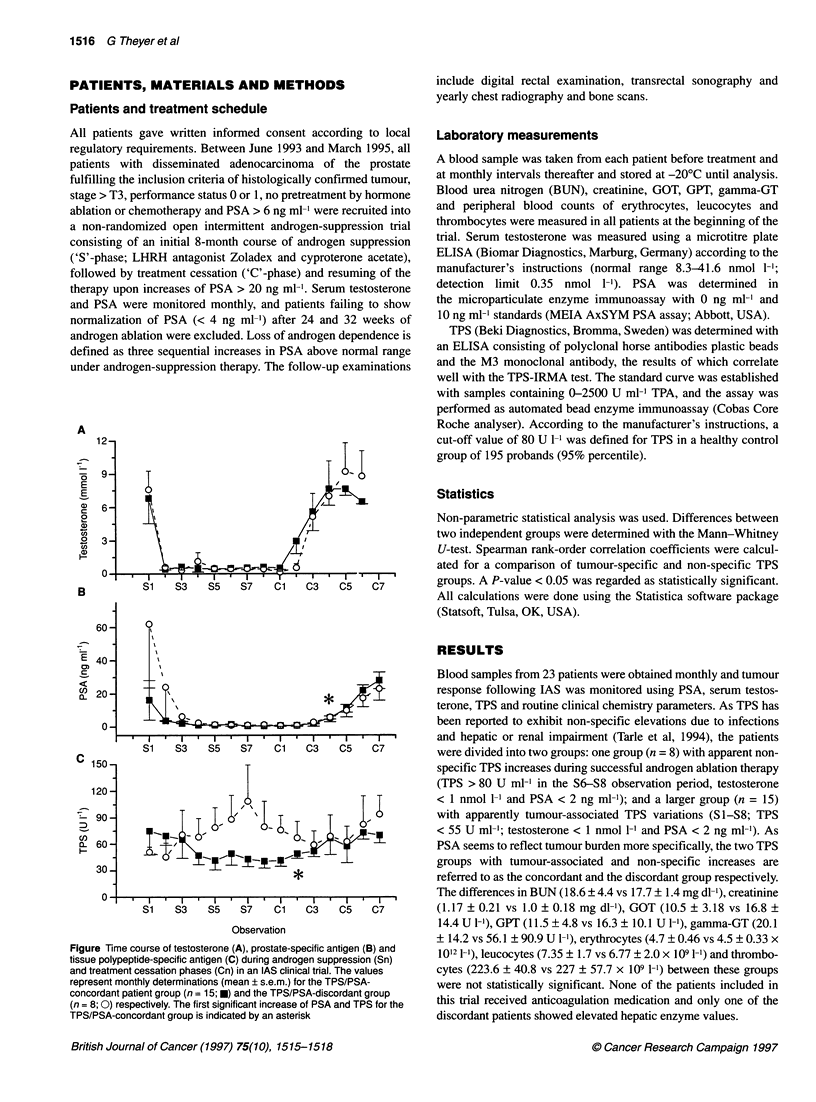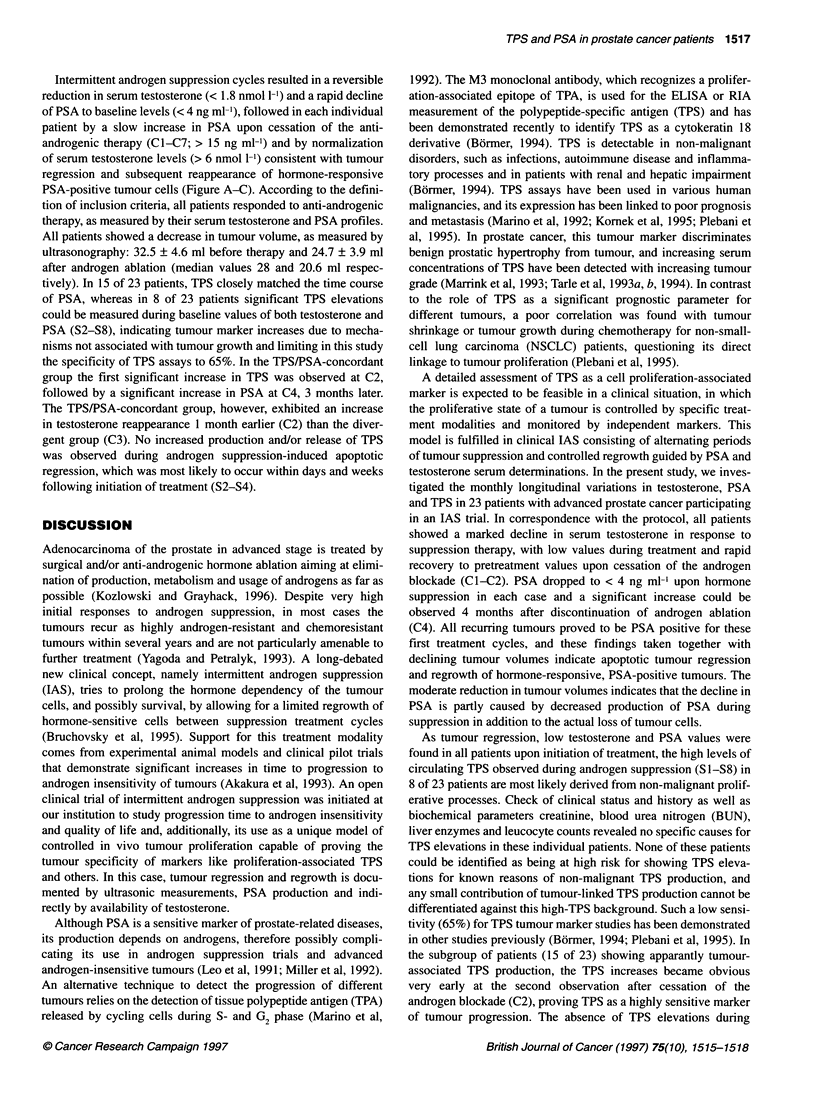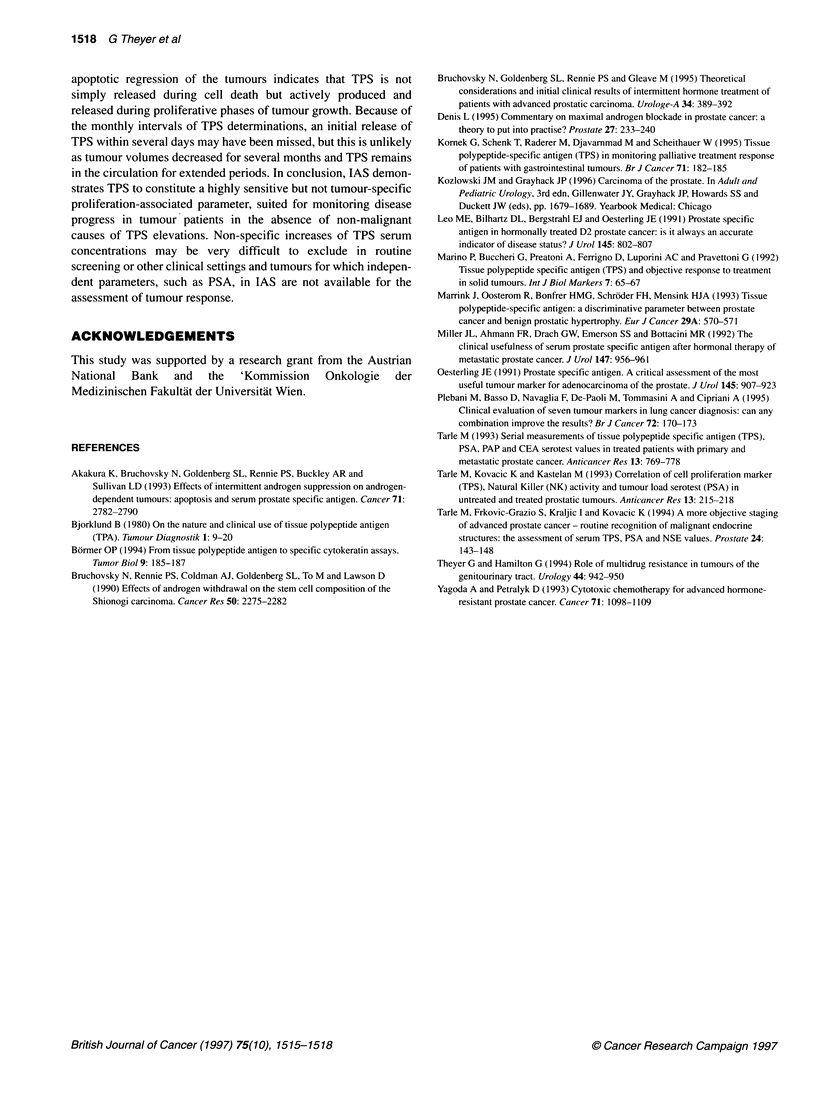# Measurements of tissue polypeptide-specific antigen and prostate-specific antigen in prostate cancer patients under intermittent androgen suppression therapy.

**DOI:** 10.1038/bjc.1997.259

**Published:** 1997

**Authors:** G. Theyer, S. Holub, A. DÃ¼rer, S. Andert, I. Haberl, U. Theyer, G. Hamilton

**Affiliations:** Department of Urology, Wilhelminenspital der Stadt Wien, Vienna, Austria.

## Abstract

The present study evaluated serial serum measurements of tissue polypeptide-specific antigen (TPS) in comparison with prostate-specific antigen (PSA) for assessment of tumour progression in patients with advanced prostate cancer receiving intermittent androgen suppression therapy (IAS). Twenty-three men were recruited into an IAS trial consisting of an initial 8 months of androgen suppression, followed by cycles of treatment cessation and resumption of therapy upon increases of PSA > 20 ng ml(-1) to prolong the hormone responsiveness of the tumour cells. Periods of androgen suppression resulted in reversible reduction in serum testosterone (< 1.8 nmol I(-1)) and PSA (< 4 ng ml(-1)) and decreases in tumour volume (mean reduction for first cycle 24 +/- 10%), indicating partial growth arrest and apoptotic regression of the tumours. In contrast to PSA values, non-specifically elevated TPS values were found in 8 of 23 patients. In 15 of 23 patients, TPS fell during periods of apoptotic tumour regression and increased simultaneously with testosterone and preceded the increases in PSA by 2 months during the period of treatment cessation. Although TPS represents a highly sensitive marker of tumour proliferation in this IAS clinical model of controlled tumour regression and regrowth, its low specificity compared with PSA limits its usefulness to monitoring of prostate cancer patients with proven absence of non-specific elevations of this marker.


British Journal of Cancer (1997) 75(10), 1515–1518
© 1997 Cancer Research Campaign

### Measurements of tissue polypeptide-specific antigen and prostate-specific antigen in prostate cancer patients under intermittent androgen suppression therapy

**G Theyer[1], S Holub[1], A Dürer[1], S Andert[2], I Haberl[3], U Theyer[3] and G Hamilton[4]**

[1]Department of Urology and [2]Department of Laboratory Medicine, Wilhelminenspital der Stadt Wien; [3]Society of Research and Treatment of Tumour Diseases and [4]Ludwig Boltzmann Institute of Clinical Oncology, KH Lainz, Vienna, Austria

**Summary** The present study evaluated serial serum measurements of tissue polypeptide-specific antigen (TPS) in comparison with prostate-specific antigen (PSA) for assessment of tumour progression in patients with advanced prostate cancer receiving intermittent androgen suppression therapy (IAS). Twenty-three men were recruited into an IAS trial consisting of an initial 8 months of androgen suppression, followed by cycles of treatment cessation and resumption of therapy upon increases of PSA > 20 ng ml$^{-1}$ to prolong the hormone responsiveness of the tumour cells. Periods of androgen suppression resulted in reversible reduction in serum testosterone (< 1.8 nmol l$^{-1}$) and PSA (< 4 ng ml$^{-1}$) and decreases in tumour volume (mean reduction for first cycle 24 ± 10%), indicating partial growth arrest and apoptotic regression of the tumours. In contrast to PSA values, non-specifically elevated TPS values were found in 8 of 23 patients. In 15 of 23 patients, TPS fell during periods of apoptotic tumour regression and increased simultaneously with testosterone and preceded the increases in PSA by 2 months during the period of treatment cessation. Although TPS represents a highly sensitive marker of tumour proliferation in this IAS clinical model of controlled tumour regression and regrowth, its low specificity compared with PSA limits its usefulness to monitoring of prostate cancer patients with proven absence of non-specific elevations of this marker.

**Keywords:** tissue polypeptide-specific antigen; prostate-specific antigen; prostate cancer; intermittent androgen suppression; tumour marker

Prostate cancer has become the most common newly diagnosed cancer in men in recent years (Kozlowski and Grayhack, 1996). When there is tumour involvement outside the prostatic capsule, this disease is ultimately incurable in most cases. Palliative treatment consists of hormonal manipulation by orchidectomy or oestrogens, LHRH analogues and steroidal or non-steroidal anti-androgens to deprive the cancer cells of androgenic stimulation (Kozlowski and Grayhack, 1996). After primary hormonal ablation by orchidectomy, the concept of maximal androgen blockade by combining anti-androgen and LHRH analogues has been propagated as means to improve survival of patients with disseminated prostate cancer (Denis, 1995). The androgen-dependent tumour cells exhibit apoptotic regression in a high percentage of cases upon this treatment. Unfortunately, this high initial response is temporary because surviving tumour cells progress to an androgen-independent growth condition (Bruchovsky et al, 1990). All attempts of a cytotoxic therapy for these androgen-resistant tumours have been met with limited success and have failed to result in significant prolongation of the low survival rates (Yagoda and Petralyk, 1993; Theyer and Hamilton, 1994).

*Received 24 June 1996*
*Revised 29 October 1996*
*Accepted 14 November 1996*

*Correspondence to:* G Theyer, Department of Urology, Wilhelminenspital der Stadt Wien, Montleartstrasse 37, A-1160 Vienna, Austria

A new clinical concept relies on the possibility of intermittent androgen suppression (IAS) to keep tumorigenic stem cells in an androgen-responsive state. The regrowing tumours have been shown to respond to androgen withdrawal for several cycles and IAS has been shown to result in improved quality of life and possible prolonged survival in pilot clinical trials (Akakura et al, 1993). Monitoring of serum testosterone and prostate-specific antigen (PSA) has been used in experimental animal models and clinical studies for the determination of the androgen-free treatment period by defining the level of tumour regrowth. However, the expression of PSA is androgen dependent, and therefore this marker may not adequately reflect tumour mass during androgen withdrawal therapy (Miller et al, 1992).

In the present study, we have investigated the tumour marker tissue polypeptide-specific antigen (TPS) in comparison with serum testosterone and PSA in a group of patients receiving IAS for assessment of the onset of tumour regrowth. The TPS assay involves the use of the M3 monoclonal antibody, recognizing a cell proliferation-associated epitope of the tissue polypeptide antigen (TPA; Börmer, 1994). TPA and TPS have been described as useful markers for a range of different tumours and in particular TPS as a sensitive indicator of tumour response (Bjorklund, 1980). IAS constitutes a clinical model of controlled apoptotic tumour regression and regrowth, and TPS can be tested for its possible correlation with proliferation/tumour cell damage in comparison to responses observed in testosterone, PSA and tumour volume.

### PATIENTS, MATERIALS AND METHODS

### Patients and treatment schedule

All patients gave written informed consent according to local regulatory requirements. Between June 1993 and March 1995, all patients with disseminated adenocarcinoma of the prostate fulfilling the inclusion criteria of histologically confirmed tumour, stage > T3, performance status 0 or 1, no pretreatment by hormone ablation or chemotherapy and PSA > 6 ng $ml^{-1}$ were recruited into a non-randomized open intermittent androgen-suppression trial consisting of an initial 8-month course of androgen suppression ('S'-phase; LHRH antagonist Zoladex and cyproterone acetate), followed by treatment cessation ('C'-phase) and resuming of the therapy upon increases of PSA > 20 ng $ml^{-1}$. Serum testosterone and PSA were monitored monthly, and patients failing to show normalization of PSA (< 4 ng $ml^{-1}$) after 24 and 32 weeks of androgen ablation were excluded. Loss of androgen dependence is defined as three sequential increases in PSA above normal range under androgen-suppression therapy. The follow-up examinations include digital rectal examination, transrectal sonography and yearly chest radiography and bone scans.

**Figure** Time course of testosterone (**A**), prostate-specific antigen (**B**) and tissue polypeptide-specific antigen (**C**) during androgen suppression (Sn) and treatment cessation phases (Cn) in an IAS clinical trial. The values represent monthly determinations (mean ± s.e.m.) for the TPS/PSA-concordant patient group (*n* = 15; ■) and the TPS/PSA-discordant group (*n* = 8; ○) respectively. The first significant increase of PSA and TPS for the TPS/PSA-concordant group is indicated by an asterisk

### Laboratory measurements

A blood sample was taken from each patient before treatment and at monthly intervals thereafter and stored at –20°C until analysis. Blood urea nitrogen (BUN), creatinine, GOT, GPT, gamma-GT and peripheral blood counts of erythrocytes, leucocytes and thrombocytes were measured in all patients at the beginning of the trial. Serum testosterone was measured using a microtitre plate ELISA (Biomar Diagnostics, Marburg, Germany) according to the manufacturer's instructions (normal range 8.3–41.6 nmol $l^{-1}$; detection limit 0.35 nmol $l^{-1}$). PSA was determined in the microparticulate enzyme immunoassay with 0 ng $ml^{-1}$ and 10 ng $ml^{-1}$ standards (MEIA AxSYM PSA assay; Abbott, USA).

TPS (Beki Diagnostics, Bromma, Sweden) was determined with an ELISA consisting of polyclonal horse antibodies plastic beads and the M3 monoclonal antibody, the results of which correlate well with the TPS-IRMA test. The standard curve was established with samples containing 0–2500 U $ml^{-1}$ TPA, and the assay was performed as automated bead enzyme immunoassay (Cobas Core Roche analyser). According to the manufacturer's instructions, a cut-off value of 80 U $l^{-1}$ was defined for TPS in a healthy control group of 195 probands (95% percentile).

### Statistics

Non-parametric statistical analysis was used. Differences between two independent groups were determined with the Mann–Whitney *U*-test. Spearman rank-order correlation coefficients were calculated for a comparison of tumour-specific and non-specific TPS groups. A *P*-value < 0.05 was regarded as statistically significant. All calculations were done using the Statistica software package (Statsoft, Tulsa, OK, USA).

### RESULTS

Blood samples from 23 patients were obtained monthly and tumour response following IAS was monitored using PSA, serum testosterone, TPS and routine clinical chemistry parameters. As TPS has been reported to exhibit non-specific elevations due to infections and hepatic or renal impairment (Tarle et al, 1994), the patients were divided into two groups: one group (*n* = 8) with apparent non-specific TPS increases during successful androgen ablation therapy (TPS > 80 U $ml^{-1}$ in the S6–S8 observation period, testosterone < 1 nmol $l^{-1}$ and PSA < 2 ng $ml^{-1}$); and a larger group (*n* = 15) with apparently tumour-associated TPS variations (S1–S8; TPS < 55 U $ml^{-1}$; testosterone < 1 nmol $l^{-1}$ and PSA < 2 ng $ml^{-1}$). As PSA seems to reflect tumour burden more specifically, the two TPS groups with tumour-associated and non-specific increases are referred to as the concordant and the discordant group respectively. The differences in BUN (18.6 ± 4.4 vs 17.7 ± 1.4 mg $dl^{-1}$), creatinine (1.17 ± 0.21 vs 1.0 ± 0.18 mg $dl^{-1}$), GOT (10.5 ± 3.18 vs 16.8 ± 14.4 U $l^{-1}$), GPT (11.5 ± 4.8 vs 16.3 ± 10.1 U $l^{-1}$), gamma-GT (20.1 ± 14.2 vs 56.1 ± 90.9 U $l^{-1}$), erythrocytes (4.7 ± 0.46 vs 4.5 ± 0.33 × $10^{12}$ $l^{-1}$), leucocytes (7.35 ± 1.7 vs 6.77 ± 2.0 × $10^9$ $l^{-1}$) and thrombocytes (223.6 ± 40.8 vs 227 ± 57.7 × $10^9$ $l^{-1}$) between these groups were not statistically significant. None of the patients included in this trial received anticoagulation medication and only one of the discordant patients showed elevated hepatic enzyme values.

© Cancer Research Campaign 1997

Intermittent androgen suppression cycles resulted in a reversible reduction in serum testosterone (< 1.8 nmol $l^{-1}$) and a rapid decline of PSA to baseline levels (< 4 ng $ml^{-1}$), followed in each individual patient by a slow increase in PSA upon cessation of the anti-androgenic therapy (C1–C7; > 15 ng $ml^{-1}$) and by normalization of serum testosterone levels (> 6 nmol $l^{-1}$) consistent with tumour regression and subsequent reappearance of hormone-responsive PSA-positive tumour cells (Figure A–C). According to the definition of inclusion criteria, all patients responded to anti-androgenic therapy, as measured by their serum testosterone and PSA profiles. All patients showed a decrease in tumour volume, as measured by ultrasonography: 32.5 ± 4.6 ml before therapy and 24.7 ± 3.9 ml after androgen ablation (median values 28 and 20.6 ml respectively). In 15 of 23 patients, TPS closely matched the time course of PSA, whereas in 8 of 23 patients significant TPS elevations could be measured during baseline values of both testosterone and PSA (S2–S8), indicating tumour marker increases due to mechanisms not associated with tumour growth and limiting in this study the specificity of TPS assays to 65%. In the TPS/PSA-concordant group the first significant increase in TPS was observed at C2, followed by a significant increase in PSA at C4, 3 months later. The TPS/PSA-concordant group, however, exhibited an increase in testosterone reappearance 1 month earlier (C2) than the divergent group (C3). No increased production and/or release of TPS was observed during androgen suppression-induced apoptotic regression, which was most likely to occur within days and weeks following initiation of treatment (S2–S4).

### DISCUSSION

Adenocarcinoma of the prostate in advanced stage is treated by surgical and/or anti-androgenic hormone ablation aiming at elimination of production, metabolism and usage of androgens as far as possible (Kozlowski and Grayhack, 1996). Despite very high initial responses to androgen suppression, in most cases the tumours recur as highly androgen-resistant and chemoresistant tumours within several years and are not particularly amenable to further treatment (Yagoda and Petralyk, 1993). A long-debated new clinical concept, namely intermittent androgen suppression (IAS), tries to prolong the hormone dependency of the tumour cells, and possibly survival, by allowing for a limited regrowth of hormone-sensitive cells between suppression treatment cycles (Bruchovsky et al, 1995). Support for this treatment modality comes from experimental animal models and clinical pilot trials that demonstrate significant increases in time to progression to androgen insensitivity of tumours (Akakura et al, 1993). An open clinical trial of intermittent androgen suppression was initiated at our institution to study progression time to androgen insensitivity and quality of life and, additionally, its use as a unique model of controlled in vivo tumour proliferation capable of proving the tumour specificity of markers like proliferation-associated TPS and others. In this case, tumour regression and regrowth is documented by ultrasonic measurements, PSA production and indirectly by availability of testosterone.

Although PSA is a sensitive marker of prostate-related diseases, its production depends on androgens, therefore possibly complicating its use in androgen suppression trials and advanced androgen-insensitive tumours (Leo et al, 1991; Miller et al, 1992). An alternative technique to detect the progression of different tumours relies on the detection of tissue polypeptide antigen (TPA) released by cycling cells during S- and $G_2$ phase (Marino et al, 1992). The M3 monoclonal antibody, which recognizes a proliferation-associated epitope of TPA, is used for the ELISA or RIA measurement of the polypeptide-specific antigen (TPS) and has been demonstrated recently to identify TPS as a cytokeratin 18 derivative (Börmer, 1994). TPS is detectable in non-malignant disorders, such as infections, autoimmune disease and inflammatory processes and in patients with renal and hepatic impairment (Börmer, 1994). TPS assays have been used in various human malignancies, and its expression has been linked to poor prognosis and metastasis (Marino et al, 1992; Kornek et al, 1995; Plebani et al, 1995). In prostate cancer, this tumour marker discriminates benign prostatic hypertrophy from tumour, and increasing serum concentrations of TPS have been detected with increasing tumour grade (Marrink et al, 1993; Tarle et al, 1993*a*, *b*, 1994). In contrast to the role of TPS as a significant prognostic parameter for different tumours, a poor correlation was found with tumour shrinkage or tumour growth during chemotherapy for non-small-cell lung carcinoma (NSCLC) patients, questioning its direct linkage to tumour proliferation (Plebani et al, 1995).

A detailed assessment of TPS as a cell proliferation-associated marker is expected to be feasible in a clinical situation, in which the proliferative state of a tumour is controlled by specific treatment modalities and monitored by independent markers. This model is fulfilled in clinical IAS consisting of alternating periods of tumour suppression and controlled regrowth guided by PSA and testosterone serum determinations. In the present study, we investigated the monthly longitudinal variations in testosterone, PSA and TPS in 23 patients with advanced prostate cancer participating in an IAS trial. In correspondence with the protocol, all patients showed a marked decline in serum testosterone in response to suppression therapy, with low values during treatment and rapid recovery to pretreatment values upon cessation of the androgen blockade (C1–C2). PSA dropped to < 4 ng $ml^{-1}$ upon hormone suppression in each case and a significant increase could be observed 4 months after discontinuation of androgen ablation (C4). All recurring tumours proved to be PSA positive for these first treatment cycles, and these findings taken together with declining tumour volumes indicate apoptotic tumour regression and regrowth of hormone-responsive, PSA-positive tumours. The moderate reduction in tumour volumes indicates that the decline in PSA is partly caused by decreased production of PSA during suppression in addition to the actual loss of tumour cells.

As tumour regression, low testosterone and PSA values were found in all patients upon initiation of treatment, the high levels of circulating TPS observed during androgen suppression (S1–S8) in 8 of 23 patients are most likely derived from non-malignant proliferative processes. Check of clinical status and history as well as biochemical parameters creatinine, blood urea nitrogen (BUN), liver enzymes and leucocyte counts revealed no specific causes for TPS elevations in these individual patients. None of these patients could be identified as being at high risk for showing TPS elevations for known reasons of non-malignant TPS production, and any small contribution of tumour-linked TPS production cannot be differentiated against this high-TPS background. Such a low sensitivity (65%) for TPS tumour marker studies has been demonstrated in other studies previously (Börmer, 1994; Plebani et al, 1995). In the subgroup of patients (15 of 23) showing apparantly tumour-associated TPS production, the TPS increases became obvious very early at the second observation after cessation of the androgen blockade (C2), proving TPS as a highly sensitive marker of tumour progression. The absence of TPS elevations during

© Cancer Research Campaign 1997

apoptotic regression of the tumours indicates that TPS is not simply released during cell death but actively produced and released during proliferative phases of tumour growth. Because of the monthly intervals of TPS determinations, an initial release of TPS within several days may have been missed, but this is unlikely as tumour volumes decreased for several months and TPS remains in the circulation for extended periods. In conclusion, IAS demonstrates TPS to constitute a highly sensitive but not tumour-specific proliferation-associated parameter, suited for monitoring disease progress in tumour patients in the absence of non-malignant causes of TPS elevations. Non-specific increases of TPS serum concentrations may be very difficult to exclude in routine screening or other clinical settings and tumours for which independent parameters, such as PSA, in IAS are not available for the assessment of tumour response.

### ACKNOWLEDGEMENTS

This study was supported by a research grant from the Austrian National Bank and the 'Kommission Onkologie der Medizinischen Fakultät der Universität Wien.

### REFERENCES

Akakura K, Bruchovsky N, Goldenberg SL, Rennie PS, Buckley AR and Sullivan LD (1993) Effects of intermittent androgen suppression on androgen-dependent tumours: apoptosis and serum prostate specific antigen. *Cancer* **71**: 2782–2790

Bjorklund B (1980) On the nature and clinical use of tissue polypeptide antigen (TPA). *Tumour Diagnostik* **1**: 9–20

Börmer OP (1994) From tissue polypeptide antigen to specific cytokeratin assays. *Tumor Biol* **9**: 185–187

Bruchovsky N, Rennie PS, Coldman AJ, Goldenberg SL, To M and Lawson D (1990) Effects of androgen withdrawal on the stem cell composition of the Shionogi carcinoma. *Cancer Res* **50**: 2275–2282

Bruchovsky N, Goldenberg SL, Rennie PS and Gleave M (1995) Theoretical considerations and initial clinical results of intermittent hormone treatment of patients with advanced prostatic carcinoma. *Urologe-A* **34**: 389–392

Denis L (1995) Commentary on maximal androgen blockade in prostate cancer: a theory to put into practise? *Prostate* **27**: 233–240

Kornek G, Schenk T, Raderer M, Djavarnmad M and Scheithauer W (1995) Tissue polypeptide-specific antigen (TPS) in monitoring palliative treatment response of patients with gastrointestinal tumours. *Br J Cancer* **71**: 182–185

Kozlowski JM and Grayhack JP (1996) Carcinoma of the prostate. In *Adult and Pediatric Urology*, 3rd edn, Gillenwater JY, Grayhack JP, Howards SS and Duckett JW (eds), pp. 1679–1689. Yearbook Medical: Chicago

Leo ME, Bilhartz DL, Bergstrahl EJ and Oesterling JE (1991) Prostate specific antigen in hormonally treated D2 prostate cancer: is it always an accurate indicator of disease status? *J Urol* **145**: 802–807

Marino P, Buccheri G, Preatoni A, Ferrigno D, Luporini AC and Pravettoni G (1992) Tissue polypeptide specific antigen (TPS) and objective response to treatment in solid tumours. *Int J Biol Markers* **7**: 65–67

Marrink J, Oosterom R, Bonfrer HMG, Schröder FH, Mensink HJA (1993) Tissue polypeptide-specific antigen: a discriminative parameter between prostate cancer and benign prostatic hypertrophy. *Eur J Cancer* **29A**: 570–571

Miller JL, Ahmann FR, Drach GW, Emerson SS and Bottacini MR (1992) The clinical usefulness of serum prostate specific antigen after hormonal therapy of metastatic prostate cancer. *J Urol* **147**: 956–961

Oesterling JE (1991) Prostate specific antigen. A critical assessment of the most useful tumour marker for adenocarcinoma of the prostate. *J Urol* **145**: 907–923

Plebani M, Basso D, Navaglia F, De-Paoli M, Tommasini A and Cipriani A (1995) Clinical evaluation of seven tumour markers in lung cancer diagnosis: can any combination improve the results? *Br J Cancer* **72**: 170–173

Tarle M (1993) Serial measurements of tissue polypeptide specific antigen (TPS), PSA, PAP and CEA serotest values in treated patients with primary and metastatic prostate cancer. *Anticancer Res* **13**: 769–778

Tarle M, Kovacic K and Kastelan M (1993) Correlation of cell proliferation marker (TPS), Natural Killer (NK) activity and tumour load serotest (PSA) in untreated and treated prostatic tumours. *Anticancer Res* **13**: 215–218

Tarle M, Frkovic-Grazio S, Kraljic I and Kovacic K (1994) A more objective staging of advanced prostate cancer – routine recognition of malignant endocrine structures: the assessment of serum TPS, PSA and NSE values. *Prostate* **24**: 143–148

Theyer G and Hamilton G (1994) Role of multidrug resistance in tumours of the genitourinary tract. *Urology* **44**: 942–950

Yagoda A and Petralyk D (1993) Cytotoxic chemotherapy for advanced hormone-resistant prostate cancer. *Cancer* **71**: 1098–1109

© Cancer Research Campaign 1997